# Synergetic Antimicrobial Effect of Silver Nanoparticles Conjugated with Iprodione against *Valsa mali*

**DOI:** 10.3390/ma15155147

**Published:** 2022-07-25

**Authors:** Tao Li, Weidong Huang, Haibing Yu

**Affiliations:** 1 College of Resources and Environment, Anhui Science and Technology University, Donghua Road 9, Chuzhou 233100, China; lit@ahstu.edu.cn; 2 College of Agriculture, Anhui Science and Technology University, Donghua Road 9, Chuzhou 233100, China

**Keywords:** silver nanoparticles, synergetic antimicrobial effect, iprodione, *Valsa* *mali*

## Abstract

Apple tree canker induced by *Valsa* *mali* is a vital disease in apple production around the world, and it highlyimpacts the development of apple industry. It is of great significance to study the inhibition effect of common fungicides and develop new fungistats for comprehensive control of apple tree canker. In this experiment, the inhibition activity of five fungicides, including mancozeb, metalaxyl, iprodione, prochloraz, and difenoconazole along with biosynthesized nanosilver against *V. mali*, were measured with the mycelium growth rate and agar well diffusion methods. The results showed that iprodione exhibited the best inhibitory effect, the median inhibition concentration (IC_50_) of iprodione and nanosilver was 0.62 μg.mL^−1^ and 45.50 μg.mL^−1^, the suppression rate achieved 67.93% at 200 μg.mL^−1^ of nanosilver. Moreover, a remarkable additive and synergistic antimicrobial effect was verified when silver nanoparticles were conjugated with iprodione at 9:1, 8:2, 7:3, and 6:4 (*v*/*v*), and the toxicity ratio was 1.04, 1.13, 1.01, and 0.98, respectively. It is proven that biosynthesized silver nanoparticles could effectively inhibit *Valsa* *mali*, and it is possible to develop and screen silver nanoparticle-based nano pesticides to manage plant diseases synthetically.

## 1. Introduction

The apple tree canker induced by *Valsa* *mali* is the most devastating disease of the apple tree, and it is a great threat to the global apple industry [1,2]. Researchers have made a huge effort to decrease its hazards, and chemical control was proved to be the most direct and frequently used among these strategies. Chemical fungicides such as asomate [3], benzamide derivatives [4], thiosemicarbazide derivatives [5], and coumarin derivatives [6] were applied for a long time. However, there exist increasing concerns in consideration of the adverse effects caused by chemical fungicides, such as resistance, residue, resurgence, environment pollution, and so on [7]. In addition, the pathogen of *V. mali* can extensively penetrate into the host’s phloem and xylem, and it is difficult for traditional chemical agents to access [8], so more environmentally friendly and efficient novel approaches like biocontrol and other ones need to be developed.

Biocontrol agents have been reported to resolve such problems induced by chemical fungicides. Tobacco cembranoids separated from tobacco flower extract can destroy the endometrial structure of the fungus of *V. mali*, and it could be totally inhibited at 80 µg.mL^−1^, and the EC_50_ value was 13.18 µg.mL^−1^ [9]. *Trichoderma longibrachiatum* T6 exhibited significant antifungal effect against *V. mali*, the inhibitory rate achieved 95% after 5 days’ incubation, and the main mechanism should be the secondary metabolites with effective bioactive substance [10]. *Bacillus velezensis* D4 showed high efficacy on *V. mali*, it could suppress the mycelial growth, and cause hyphal damage [11]. Although biocontrol agents exhibit lower toxicity and wider source compared with chemical fungicides, several disadvantages such as poor stability, environmental sensitivity, and high cost have been increasingly emerging. It is urgent to develop novel approaches to resolve such problems.

Fortunately, versatile nanotechnology has emerged and infiltrated in multiple areas including drug delivery, optics, chemistry, biology, etc. Extremely fine nanomaterials exhibit unique and distinguished properties compared with their bulk counterparts [12,13,14,15]. There are three main approaches from physics, chemistry, and biology to synthesize nanomaterials. Synthesis of metal nanomaterials such as Silver, CuO, etc., were reported by Yi [16] and Khatami [17]. Application of some nonmetallic nanomaterials including polymeric, lipid, etc., were summarized by Zazo [18] and Rajwade [19]. In addition, many other single [20,21,22,23,24] and compound [25,26,27] nanomaterials were also synthesized and applied by different researchers. Silver nanoparticles stand out from these nanomaterials owing to their prominent inhibition activity against various pathogens. A large number of living bodies such as microorganisms [28,29,30,31] and plant tissues [32,33,34,35] were used to biosynthesize silver nanoparticles, and their antimicrobial effects against different pathogens were also determined by researchers. Because of the adverse influences caused by chemical agents, it is urgent to decrease their dosage without reducing inhibition efficiency. It was reported that silver nanoparticles could be combined with antibiotics [36,37,38] and fungicides [39,40,41] to achieve synergistic antibacterial and antifungal effect against general pathogens; moreover, the conjugations could even show excellent synergistic activity against multi-drug resistant strain [42,43].

In this report, the sensitivity of *V. mali* to five general fungicides was determined, and the most sensitive fungicide was identified: it will provide a reference for field disease control. A traditional Chinese herbal medicine called *Trachycarpus fortunei* that has the effect of astringency and hemostasis was applied to synthesize silver nanoparticles for the first time. Green synthesis of nanoparticles based on plant tissues expresses several advantages such as abundant raw materials, low synthetic cost, low energy, and no external additives compared with traditional physical and chemical approaches. Moreover, it also can reduce synthesis time compared with biosynthesis by microorganisms. As far as we know, it is the first time to apply mycelium growth and agar well diffusion methods to evaluate the antifungal activity of biosynthesized silver nanoparticles against *V. mali*, and the synergistic antifungal effect of silver nanoparticles and iprodione were also conducted. The results would provide a novel approach to integrative control of *V. mali*, and it also has important significance for decreasing dosage of chemical pesticides and enhancing inhibitory efficiency.

## 2. Materials and Methods

### 2.1. Fungicides and Isolate

*T. fortune* leaves (Fengyang, China), *V. mali* (Fengyang, China), and AgNO_3_ (Sinopharm Chemical Reagent Co., Ltd., Shanghai, China) were preserved at plant protection laboratory, Anhui Science and Technology University. The concentration of five fungicides is illustrated in Table 1.

### 2.2. Determination of Fungicides Sensitivity against V. mali

The stock solution of five fungicides was 10 mg.mL^−1^, a concentration gradient that containedPDA (Potato dextrose agar, Sinopharm Chemical Reagent Co., Ltd., Shanghai, China) plate is shown in Table 1. Blocks of *V. mali* were drilled by a sterile hole puncher (φ = 8 mm) from fungus cultured for seven days. A strain disk was transferred in the middle of each fungicide contained PDA plate, the plates that contained sterile water were set as control, then incubated at 28 °C for 5–7 d.

### 2.3. Biosynthesis and Characterization of Nanoparticles

About 100 mL deionized water was combined with 10 g *T. fortunei* dry leaf powders, which was heated at 100 °C for 20 min. The plant extract was filtrated by a millipore filter (φ = 0.22 μm). For green synthesis of silver nanoparticles, 5 mL leaf filtrate and 1 mmol. L^−1^ AgNO_3_ were added to deionized water, the process of heating at 80 °C did not stopped until the color changed. UV-vis spectroscopy, TEM, XRD, and AFM were adopted to characterize synthesized nanoparticles.

### 2.4. Fungus Growth Influence

About 5 mL silver nanoparticles and sterile water were added to 45 mL PDA medium to make the concentration of nanoparticles was 10, 25, 50, 100, and 200 μg.mL^−1^, respectively. PDA medium with 5 mL sterile water was set as control. A strain block (φ = 8 mm) was transferred to the center of each PDA medium, then incubated at 28 °C for 7 d. Inhibition rate was calculated by the following equation.


Inhibition rate (%) = [(φ_control colony_ − φ_treatment colony_)/(φ_control colony_ − φ_fungus block_)] × 100%

### 2.5. Inhibition Zone Measurement

About 0.1 mL conidia suspension of *V. mali* was spread on PDA medium. Agar wells were made and equally distributed on it. Afterwards, 30 μL different concentrations (2 and 5 μg.mL^−1^) of iprodione and silver nanoparticles (100 and 200 μg.mL^−1^) were dripped into the wells, and sterile water (30 μL) was set as control. Inhibition zone diameter was obtained after 48–72 h.

### 2.6. Leakage of DNA and Protein

The antifungal activity of silver nanoparticles against *V. mali* was also measured in terms of leakage of DNA and protein referred to previous reports [20,44]. The spore suspension was prepared as above and silver nanoparticles were mixed at the concentration of 0, 10, 25, 50, 100, and 200 μg.mL^−1^. After incubating at 28 °C for 48 h, the leakage of DNA and protein contents from *V. mali* were measured by assaying absorbance at 260 nm (A_260_) and 280 nm (A_280_) through UV-vis spectrometer.

### 2.7. Synergistic Inhibition Effect of Silver Nanoparticles and Iprodione

The IC_50_ of nanoparticles and iprodione was determined by colony growth inhibition method. The proportion of the two was 0:10, 1:9, 2:8, 3:7, 4:6, 5:5, 6:4, 7:3, 8:2, 9:1, and 10:0 (*v*/*v*), respectively. The synergistic effect assessment (toxicity ratio) of silver nanoparticles and iprodione was counted by the reference [41].

## 3. Results

### 3.1. Sensitivity of Fungicides against V. mali

The toxicity of five fungicides against *V. mali* showed a significant difference (Table 2), the IC_50_ was in the range of 0.62–54.71 μg.mL^−1^, and the 95% confidence limit was between 0.39 and 85.83 μg.mL^−1^. Iprodione, peochloraz, and difenoconazole were identified as highly sensitive fungicides; however, mancozeb and metalaxyl were identified as insensitive ones. The result will provide guidance for choosing efficient fungicide to inhibit *V. mali*. Therefore, the most sensitive fungicide of iprodione was chosen to conjugate with silver nanoparticles to determine their synergistic activity.

### 3.2. Biosynthesis of Silver Nanoparticles

**Figure 1:**
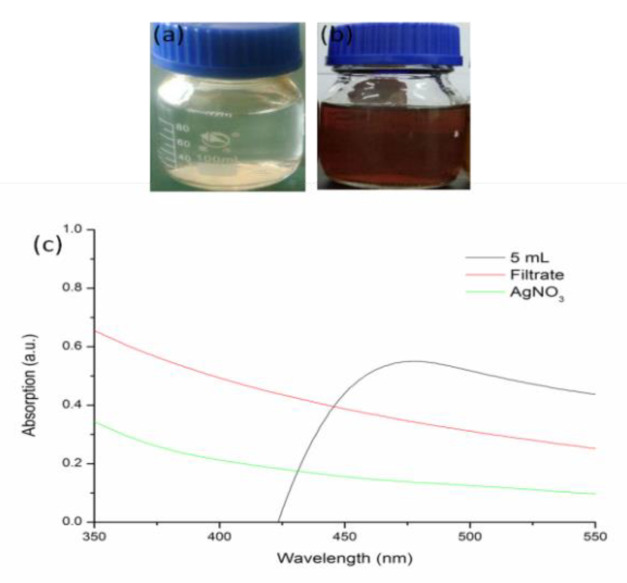
Green synthesis of silver nanoparticles by *T. fortunei* leaf extract. (**a**) leaf filtrate without AgNO_3_; (**b**) leaf filtrate with AgNO_3_; (**c**) UV-vis absorption spectrum.

The solution contained T. *fortune* leaf filtrate and AgNO_3_ changed into dark brown after heating at 80 °C for 15 min (Figure 1b), while it kept light yellow as there was no AgNO_3_ in the leaf extract (Figure 1a). A strong signal appeared at 462 nm scanned by UV-vis spectroscope, indicating the characteristic absorption peak of silver nanoparticles [45,46] that is different from leaf extract or AgNO_3_ alone (Figure 1c).

### 3.3. Characterization

#### 3.3.1. TEM Analysis

**Figure 2:**
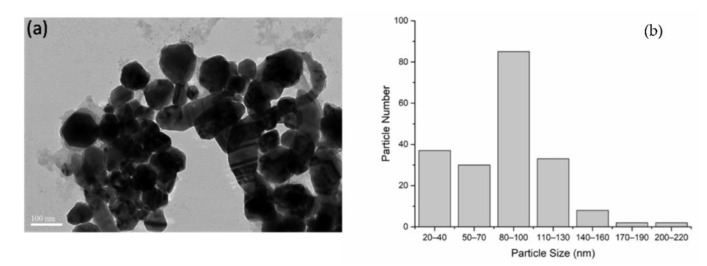
TEM image (**a**) and size distribution (**b**) of silver nanoparticles.

May kinds of plants were used to biosynthesize silver nanoparticles, and the morphology of synthesized nanoparticles varied, the most common one was spherical or near spherical. As shown in Figure 2a, nanoparticles that synthesized by *T.* *fortunei* leaf extract were polygonal or irregular in shape, the reasons that caused this variance could be different plant species, varied synthesis parameters, etc. The particle diameter was between 27 and 223 nm, and the average diameter was about 88 nm (Figure 2b).

#### 3.3.2. XRD Measurement

**Figure 3:**
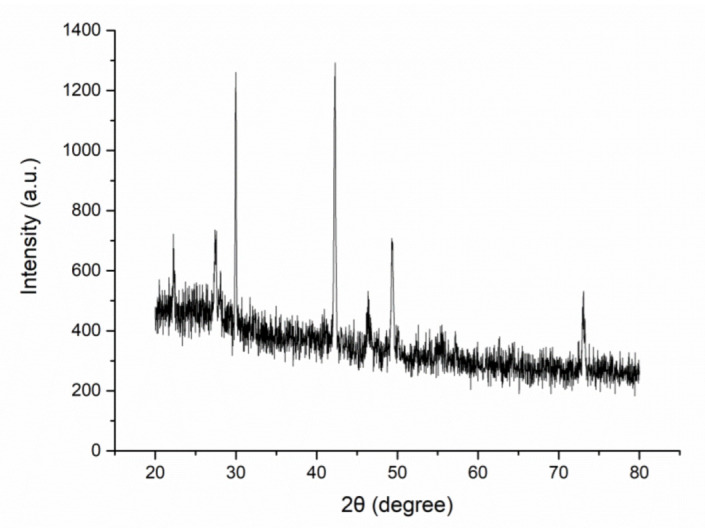
XRD pattern of silver nanoparticles.

Figure 3 showed the XRD pattern of synthesized silver nanoparticles, it indicates the existence of silver with a monoclinic crystalline system. The 2θ values of 22.24°, 27.56°, 28.06°, 29.96°, 42.28°, 46.36°, and 49.30° on it might be classified as the silver faces of (111), (200), and (220) [47], the 2θ value of 73.08° might belonged to (420) plane [48].

#### 3.3.3. AFM Analysis

**Figure 4:**
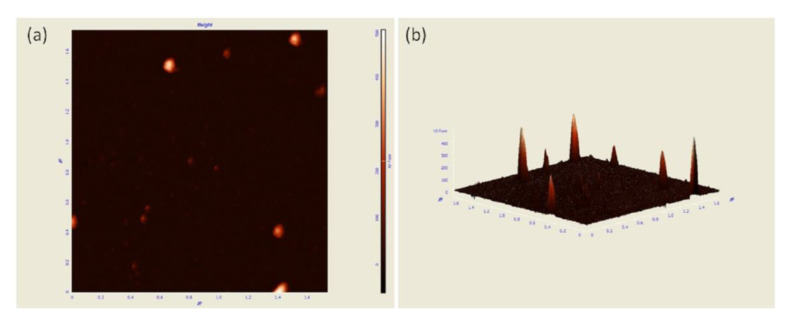
AFM image of silver nanoparticles. Morphological characteristic (**a**) and 3D topographic image (**b**) of silver nanoparticles.

The specific morphological characteristic of the biosynthesized silver nanoparticles was detected by AFM. The particles deposited on the substrate dispersed well (Figure 4a), and the 3D topographic image was also presented as Figure 4b.

### 3.4. Antifungal Activity of Silver Nanoparticles

#### 3.4.1. Colony Growth Inhibition

**Figure 5:**
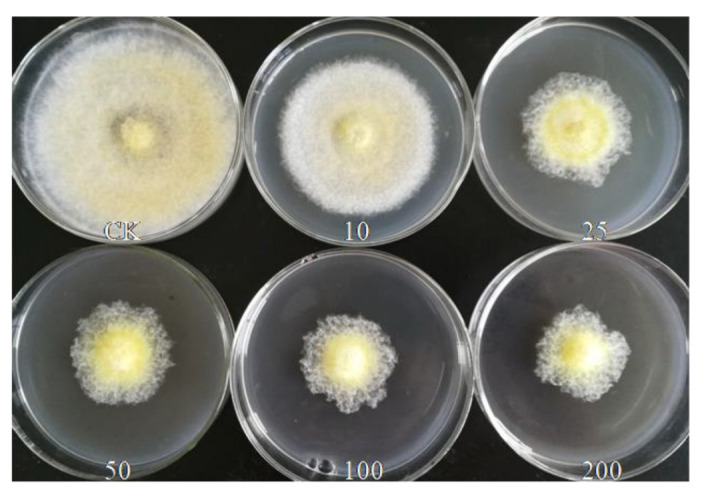
Colony growth inhibition of silver nanoparticles against *V. mali*.

Various concentrations of silver nanoparticles displayed obvious inhibition effects against *V. mali* and they were positively correlated with the concentration. As shown in Figure 5, the diameter of control was 9.0 cm, the IC_50_ of silver nanoparticles was 45.5 μg.mL^−1^, the 95% confidence limit was in the range of 12.47–140.75 μg.mL^−1^, when the concentration of silver nanoparticles enhanced to 200 μg.mL^−1^, it achieved its minimum value of 3.43 cm, and the inhibition rate was 67.93%.

#### 3.4.2. Inhibition Zone Diameter

The inhibition zone diameter was determined by the agar well diffusion approach. Table 3 showed that inhibition zone diameter varied with different fungisats and concentrations. For the control, no inhibition zone appeared near the agar well, while for iprodione and silver nanoparticles, an obvious inhibition zone was created near the agar wells. When the concentration of iprodione enhanced from 2 to 5 μg.mL^−1^, the diameter enlarged from 18.50 ± 1.81 to 22.30 ± 2.02 mm. When the concentration of silver nanoparticles increased from 100 to 200 μg.mL^−1^, it enlarged from 10.80 ± 1.13 to 12.50 ± 1.22 mm.

#### 3.4.3. Determination of the Leakage of DNA and Protein

**Figure 6:**
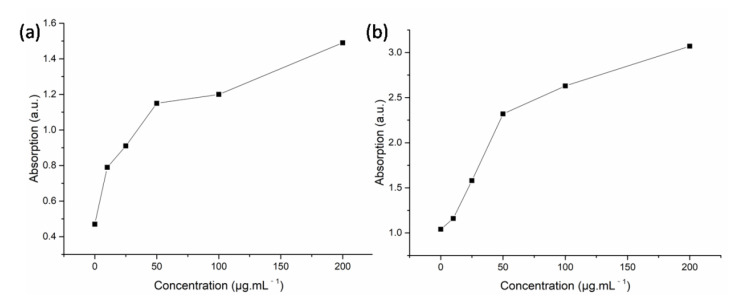
Leakage of DNA (**a**) and protein (**b**) of silver nanoparticles against *V. mali*.

DNA and protein are two important materials in any living body. Exogenous biotic or abiotic stresses, such as pathogens, insects, drought, salt, and so forth, can affect these materials. Figure 6 shows that the leakage of both DNA and protein increased dramatically with the increasing concentration of silver nanoparticles. For the leakage of DNA, the initial absorption at 260 nm (OD_260_) of the control was 0.47. When the concentration of silver nanoparticles increased from 10 to 200 μg.mL^−1^, the absorption was in the range of 0.79 and 1.49; the maximum was 3.17 times more than that of the control. For the leakage of protein, the initial absorption at 280 nm (OD_280_) of the control was 1.04. It reached 3.07 when the concentration of silver nanoparticles was 200 μg.mL^−1^, which was 2.95 times more than that of the control. It showed that the cell membrane of V. mali was interrupted by different concentrations of silver nanoparticles, and the degree of leakage had a positive correlation with the concentration of these nanoparticles. The results were similar to previous reports in which silver nanoparticles were applied to treat *Fusarium graminearum* [20], and CS-Mg nanocomposite was applied to treat *Acidovorax oryzae* and *Rhizoctonia solani* [44].

#### 3.4.4. Synergistic Antimicrobial Effect of Silver Nanoparticles Conjugated with Iprodione

The synergistic effect of biosynthesized silver nanoparticles conjugated with iprodione is shown in Table 4. The obvious synergistic antifungal activity appeared at 9:1 and 8:2, and the toxicity ratio achieved 1.04 and 1.13, respectively. The additive activity appeared at 7:3 and 6:4, and the toxicity ratio was 1.01 and 0.98. However, at other volume ratios, an antagonistic effect occurred.

## 4. Discussion

*V. mali* is a vital pathogen that causes enormous loss to apple industry. Although conventional chemical management inhibits it effectively, environmental pollution and agricultural product safety are particularly worrying [3,5,7]. With the strengthening of environmental safety awareness, more and more researchers turn their attention to biological control, multiple plant extract, biocontrol bacterium, biocontrol fungi were screened [9,11]. There is no doubt that biocontrol agents have advantages such as being eco-friendly and widely-sourced compared with chemical pesticides; however, several drawbacks such as low inhibition efficiency, high cost, and instability have come into being. As a result, more novel, higher efficient, more stable fungistats urgently need to be explored

Nanomaterials that possess unique chemical, physical, biological, and electronical characteristics could resolve such problems [13,17]. In recent years, many kinds of living bodies were used to synthesize nanoparticles through the biological approach, such as *Conyza* *Canadensis* [16], *Stachys lavandulifolia* [17], *Fusarium chlamydosporum* and *Penicillium chrysogenum* [20], *Klebsiella pneumonia* [31], etc. The morphology of these synthesized nanoparticles was near round or spherical, which are similar to our result. Such synthesized nanoparticles were also applied in the fields of cell cytotoxic or pathogen inhibition. Sarani et al., confirmed that biosynthesized α-Bi_2_O_3_ NPs, Mn-doped and Zn-doped Bi_2_O_3_ NPs showed potent cytotoxic effect against breast cancer (MCF-7) and human umbilical vein endothelial (HUVEC) cells [27]. Silver nanoparticles synthesized by a green approach expressed 50% higher antibacterial effect against foodborne pathogens compared with untreated sample [32]. Dhara et al. compared the antimicrobial activity of biosynthesized and chemically synthesized silver nanoparticles; the results showed that biosynthesized ones possess better antibacterial effects than the chemical ones against both Gram-positive and the Gram-negative bacteria [34]. For our experiment, the antimicrobial activity of silver nanoparticles synthesized by *T. fortunei* leaf extract was not quite the same compared with previous reports. It is concluded that antimicrobial effect of the same nanoparticles synthesized through the same approach or not varied against different pathogens, or different nanoparticles exhibited diverse antimicrobial activity against the same pathogen. The biosynthesis process of silver nanoparticles by *T. fortunei* leaf spent less time, and such synthesized nanoparticles exhibited multidimensional antifungal activity against *V. mali*, which showed more advantages compared with traditional inhibition approaches.

Under the new situation, increasing numbers of scholars appealed for a reduction in the dosage of chemical pesticides to improve environment quality and agricultural product safety. Hwang et al. confirmed the synergistic antibacterial effect of silver nanopartiles and three antibiotics such as ampicilin, chloramphenicol, and kanamycin against different pathogenic bacteria [36]. The conjugation of biosynthesized silver nanoparticles and Imipenem showed higher antibacterial effect against *Serratia fonticola* and *Pantoea* sp. compared with chemical synthesized nanoparticles [37]. The synergistic antibacterial effect was proved when silver nanoparticles were combined with streptomycin sulfate against Gram-negative and Gram-positive bacteria [38]. McShan et al. evaluated the synergistic antibacterial effect of silver nanoparticles and tetracycline, neomycin, and penicillin against multi-drug resistant bacterium *Salmonella typhimurium* DT104; the results showed that the antimicrobial effect could be assisted by tetracycline, neomycin, while not by penicillin [42]. There were also some fungicides and antibiotics that combined with silver nanoparticles to evaluate their synergistic activity, such as polyene antifungals against *Candida parapsilosis* [39], echinocandin and azole fungicides against *Candida albicans* [40], epoxiconazole against *Setosphaeria turcica* [41], fluconazole against *Candida albicans* [43]. The antifungal activity of silver nanoparticles and iprodione were classified as three types at different volume ratios, i.e., synergistic, additive, and antagonistic. The reason for that might be different species of silver nanoparticles and fungicides, varied evaluation methods, and so on.

## 5. Conclusions

In this research, iprodione was screened as the highest efficient chemical fungicides to inhibit *V. mali*; it will provide a reference for control Apple tree canker. *T. fortunei* leaf extract that has the effect of astringency and hemostasis can be used to biosynthesize silver nanoparticles, and the antifungal effect of silver nanoparticles and synergistic antifungal activity of silver nanoparticles and iprodione was determined for the first time. The results showed that silver nanoparticles biosynthesized by *T. fortunei* can be used to suppress this pathogen effectively. In addition, distinct synergistic activity exhibited when silver nanoparticles conjugated with iprodione at certain volume ratios, which can be a candidate to assist chemical fungicides to play their roles.

## Figures and Tables

**Table 1:** Fungicides and their concentrations.

Fungicide	Concentration Gradient (μg·mL^−1^)	Manufacturer
mancozeb 96% TC	5.0, 10.0, 20.0, 50.0, 100.0	Limin Chemical Co. LTD, Xinyi, China
metalaxyl 97% TC	0.05, 0.2, 0.5, 2.0, 5.0	Yifan Biotechnology Group Co. LTD, Wenzhou, China
iprodione 96% TC	0.05, 0.2, 0.5, 2.0, 5.0	Jiangxi Heyi Chemical Co., LTD, Jiujiang, China
prochloraz 97% TC	0.05, 0.2, 0.5, 2.0, 5.0	Jiangsu Yunfan Chemical Co., LTD, Qidong, China
difenoconazole 95% TC	10.0, 20.0, 50.0, 100.0, 200.0	Limin Chemical Co. LTD, Xinyi, China

**Table 2:** Sensitivity of five fungicides against *V.mali*.

Fungicide	Toxicity Regression	IC_50_ (μg·mL^−1^)	95% Confidence Limit (μg·mL^−1^)	R^2^
mancozeb	y = 1.26880x + 2.10396	45.52	34.09–66.62	0.708
iprodione	y = 0.75206 − 0.15605	0.62	0.39–0.99	0.856
prochloraz	y = 0.92575x − 0.00364	0.99	0.69–1.50	0.937
metalaxyl	y = 0.67065x + 1.16562	54.71	34.56–85.83	0.984
difenoconazole	y = 0.66464x − 0.18641	1.90	1.13–4.09	0.775

**Table 3:** Inhibition zone diameter of iprodione and silver nanoparticles.

Fungistat	Concentration (μg·mL^−1^)	Inhibition Zone Diameter (mm)
sterile water	/	0.00 ± 0.00
iprodione	2	18.50 ± 1.81
iprodione	5	22.30 ± 2.02
silvernanoparticles	100	10.80 ± 1.13
silvernanoparticles	200	12.50 ± 1.22

**Table 4:** Toxicity ratio of nanosilver and iprodione against *V. mali*.

Volume Ratio	Actual Inhibition Rate (%)	Theoretical Inhibition Rate (%)	Toxicity Ratio
10:0	50.83	50.83	1.00
9:1	53.33	51.25	1.04
8:2	58.33	51.67	1.13
7:3	52.67	52.08	1.01
6:4	51.67	52.50	0.98
5:5	50.00	52.92	0.94
4:6	46.67	53.33	0.88
3:7	43.83	53.75	0.82
2:8	40.50	54.17	0.75
1:9	50.00	54.58	0.92
0:1	55.00	55.00	1.00

## Data Availability

Not applicable.

## References

[1] Wang X.L., Wei J.L., Huang L.L., Kang Z.S. (2011). Re-evaluation of pathogens causing Valsa canker on apple in China. Mycologia.

[2] Wang X.L., Zang R., Yin Z.Y., Kang Z.S., Huang L.L. (2014). Delimiting cryptic pathogen species causing apple Valsa canker with multilocus data. Ecol. Evol..

[3] Cao K., Guo L., Li B., Sun G., Chen H. (2009). Investigations on the occurrence and control of apple canker in China. Plant Prot..

[4] Lei P., Xu Y., Du J., Yang X.L., Yuan H.Z., Xu G.F., Ling Y. (2016). Design, synthesis and fungicidal activity of N-substituted benzoyl-1,2,3,4-tetrahydroquinolyl-1-carboxamide. Bioorg. Med. Chem..

[5] Zhang X.B., Lei P., Sun T.D., Jin X.Y., Yang X.L., Ling Y. (2017). Design, synthesis, and fungicidal activity of novel thiosemicarbazide derivatives containing piperidine fragments. Molecules.

[6] Yan W., Wei P., Wang D., Hao S.H., Li W.W., Ding F. (2018). Design, synthesis, antifungal activity, and 3D-QSAR of coumarin derivatives. J. Pestic. Sci..

[7] Kim B.S., Hwang B.K. (2007). Microbial fungicides in the control of plant diseases. J. Phytopathol..

[8] Yin Z.Y., Liu H.Q., Li Z.P., Ke X.W., Dou D.L., Gao X.N., Song N., Dai Q.Q., Wu Y.X., Xu J.R. (2015). Genome sequence of Valsa canker pathogens uncovers a potential adaptation of colonization of woody bark. New Phytol..

[9] Duan S.Z., Du Y.M., Hou X.D., Yan N., Dong W.J., Mao X.X., Zhang Z.F. (2016). Chemical Basis of the Fungicidal Activity of Tobacco Extracts against *Valsa mali*. Molecules.

[10] Zhang S.W., Xu B.L., Zhang J.H., Gan Y.T. (2018). Identification of the antifungal activity of *Trichoderma longibrachiatum* T6 and assessment of bioactive substances in controlling phytopathgens. Pestic. Biochem. Phys..

[11] Liu R., Li J., Zhang F., Zheng D., Chang Y., Xu L., Huang L. (2021). Biocontrol activity of Bacillus velezensis D4 against apple Valsa canker. Biol. Control.

[12] Lai W.F. (2020). Non-conjugated polymers with intrinsic luminescence for drug delivery. J. Drug Deliv. Sci. Technol..

[13] Lai W.F., Tang R., Wong W.T. (2020). Ionically Crosslinked Complex Gels Loaded with Oleic Acid-Containing Vesicles for Transdermal Drug Delivery. Pharmaceutics.

[14] Bakshi M., Singh H.B., Abhilash P.C. (2014). The unseen impact of nanoparticles: More or less. Curr. Sci..

[15] Khademhosseini A., Parak W.J., Weiss P.S. (2016). Nanoscience and Nanotechnology around the World. ACS Nano.

[16] Yi Y.M., Wang C.J., Cheng X.X., Yi K.C., Huang W.D., Yu H.B. (2021). Biosynthesis of Silver Nanoparticles by *Conyza*
*canadensis* and Their Antifungal Activity against *Bipolaris maydis*. Crystals.

[17] Khatami M., Heli H., Jahani P.M. (2017). Copper/copper oxide nanoparticles synthesis using *Stachys lavandulifolia* and its antibacterial activity. IET Nanobiotechnol..

[18] Zazo H., Colino C.I., Lanao J.M. (2016). Current applications of nanoparticles in infectious diseases. J. Control. Release.

[19] Rajwade J.M., Chikte R.G., Paknikar K.M. (2020). Nanomaterials: New weapons in a crusade against phytopathogens. Appl. Microbiol. Biot..

[20] Khalil N.M., El-Ghany M.N.A., Rodríguez-Couto S. (2019). Antifungal and anti-mycotoxin efficacy of biogenic silver nanoparticles produced by *Fusarium chlamydosporum* and *Penicillium chrysogenum* at non-cytotoxic doses. Chemosphere.

[21] Huang W.D., Fang H., Zhang S.Y., Yu H.B. (2021). Optimised green synthesis of copper oxide nanoparticles and their antifungal activity. MicroNano Lett..

[22] Miri A., Najafzadeh H., Darroudi M., Miri M.J., Kouhbanani M.A.J., Sarani M. (2021). Iron Oxide Nanoparticles: Biosynthesis, Magnetic Behavior, Cytotoxic Effect. ChemistryOpen.

[23] Sharma R., Tripathi A. (2022). Green synthesis of nanoparticles and its key applications in various sectors. Mater. Today Proc..

[24] Samuel M.S., Ravikumar M., John A., Selvarajan E., Patel H., Chander P.S., Soundarya J., Vuppala S., Balaji R., Chandrasekar N. (2022). A Review on Green Synthesis of Nanoparticles and Their Diverse Biomedical and Environmental Applications. Catalysts.

[25] Shokoofeh N., Moradi-Shoeili Z., Naeemi A.S. (2019). Biosynthesis of Fe_3_O_4_@Ag nanocomposite and evaluation of its performance on expression of norA and norB efflux pump genes in ciprofloxacin-resistant *Staphylococcus aureus*. Biol. Trace Elem. Res..

[26] Qasim M., Singh B.R., Naqvi A.H., Paik P., Das D. (2015). Silver nanoparticles embedded mesoporous SiO_2_ nanosphere: An effective anticandidal agent against *Candida albicans* 077. Nanotechnology.

[27] Sarani M., Tosan F., Hasani S.A., Barani M., Adeli-sardou M., Khosravani M., Niknam S., Kouhbanani M.A.J. (2022). Study of in vitro cytotoxic performance of biosynthesized α-Bi_2_O_3_ NPs, Mn-doped and Zn-doped Bi_2_O_3_ NPs against MCF-7 and HUVEC cell lines. J. Mater. Res. Technol..

[28] Waghmare S.R., Mulla M.N., Marathe S.R., Sonawane K.D. (2015). Ecofriendly production of silver nanoparticles using *Candida utilis* and its mechanistic action against pathogenic microorganisms. Biotechnology.

[29] Sarsar V., Selwal M.K., Selwal K.K. (2016). Biogenic synthesis, optimisation and antibacterial efficacy of extracellular silver nanoparticles using novel fungal isolate *Aspergillus fumigates* MA. IET Nanobiotechnol..

[30] Anandalakshmi K. (2021). Review on biosynthesis of silver nanoparticles and its characterization. Plant Arch..

[31] Sayyid N.H., Zghair Z.R. (2021). Biosynthesis of silver nanoparticles produced by *Klebsiella pneumonia*. Mater. Today.

[32] Krishnan S., Srisrimal D., Srisrimal A.K. (2020). Antimicrobial Effectiveness of Silver Nanoparticles enriched Tea Leaves. Int. J. Pharm. Qual. Assur..

[33] Chaudhari P., Chaudhari P.M., PatilP H. (2020). Antimicrobial effects of silver nanoparticle using various Indian traditional herbs. Int. J. Adv. Res..

[34] Dhara B., Roy I., Maity A. (2021). Comparative Account of the Genotoxic and Antimicrobial Effects of Silver Nanoparticles Synthesized from Extract of *Pleurotus*
*ostreatus* and Chemically Synthesized Nanoparticles. Cell Tissue Biol..

[35] Abdul S., Kadhem S., Salman K. (2022). Antimicrobial effect of silver nanoparticles with *Kluyvera cryocrescens* and biofilm cultures. Life Sci. Arch..

[36] Hwang I.S., Hwang J.H., Choi H., Kim K.J., Lee D.G. (2012). Synergistic effects between silver nanoparticles and antibiotics and the mechanisms involved. J. Med. Microbiol..

[37] Hasson S.O., Al-Awady M.J., Al-Hamadani A.H., Al-Azawi I.H. (2019). Boosting antimicrobial activity of imipenem in combination with silver nanoparticles towards *S. fonticola* and Pantoeasp. Nano Biomed. Eng..

[38] Huang W.D., Wang J., Wang Z.X., Yu H.B. (2021). Synergistic antimicrobial activity of silver nanoparticles combined with streptomycin sulfate against gram-negative and gram-positive bacteria. Mol. Cryst. Liq. Cryst..

[39] Da Frota S.M., Cunha F.A., Cunha M.D.C.D.S.O., Martins R.T., Menezes E.A., Fechine P.B.A. (2018). Synergistic Eeffect of Polyene Antifungals and Silver Nanoparticles Against *Candida parapsilosis*. J. Antibiot. Res..

[40] Li H., Wang L.H., Chai Y.F., Cao Y.B., Lu F. (2018). Synergistic effect between silver nanoparticles and antifungal agents on *Candida albicans* revealed by dynamic surface-enhanced Raman spectroscopy. Nanotoxicology.

[41] Huang W.D., Yan M.H., Duan H.M., Bi Y.L., Cheng X.X., Yu H.B. (2020). Synergistic Antifungal Activity of Green Synthesized Silver Nanoparticles and Epoxiconazole against *Setosphaeria turcica*. J. Nanomater..

[42] McShan D., Zhang Y., Deng H., Ray P.C., Yu H.T. (2015). Synergistic Antibacterial Effect of Silver Nanoparticles Combined with Ineffective Antibiotics on Drug Resistant *Salmonella typhimurium* DT104. J. Environ. Sci. Health C.

[43] Longhi C., Santos J.P., Morey A.T., Marcato P.D., Duran N., Pinge-Filho P., Nakazato G., Yamada-Ogatta S.F., Yamauchi L.M. (2015). Combination of fluconazole with silver nanoparticles produced by *Fusarium oxysporum* improves antifungal effect against planktonic cells and biofilm of drug-resistant *Candida albicans*. Med. Mycol..

[44] Ahmed T., Noman M., Luo J.Y., Muhammad S., Shahid M., Arshad A.M., Zhang M.C., Li B. (2021). Bioengineered chitosan-magnesium nanocomposite: A novel agricultural antimicrobial agent against *Acidovorax oryzae* and *Rhizoctonia solani* for sustainable rice production. Int. J. Biol. Macromol..

[45] Ibrahim E., Zhang M., Zhang Y., Hossain A., Qiu W., Chen Y., Wang Y., Wu W., Sun G., Li B. (2020). Green-Synthesization of Silver Nanoparticles Using Endophytic Bacteria Isolated from Garlic and Its Antifungal Activity against Wheat Fusarium Head Blight Pathogen *Fusarium graminearum*. Nanomaterials.

[46] Khatami M., Mehnipor R., Poor M.H.S., Jouzani G.S. (2016). Facile Biosynthesis of Silver Nanoparticles Using *Descurainia sophia* and Evaluation of Their Antibacterial and Antifungal Properties. J. Cluster Sci..

[47] Sathiya C.K., Akilandeswari S. (2014). Fabrication and characterization of silver nanoparticles using Delonixelata leaf broth. Spectrochim. Acta A.

[48] Philip D. (2010). Green synthesis of gold and silver nanoparticles using *Hibiscus rosasinensis*. Phys. E Low Dimens. Syst. Nanostruct..

